# Discovery of chitin in skeletons of non-verongiid Red Sea demosponges

**DOI:** 10.1371/journal.pone.0195803

**Published:** 2018-05-15

**Authors:** Hermann Ehrlich, Lamiaa A. Shaala, Diaa T. A. Youssef, Sonia Żółtowska- Aksamitowska, Mikhail Tsurkan, Roberta Galli, Heike Meissner, Marcin Wysokowski, Iaroslav Petrenko, Konstantin R. Tabachnick, Viatcheslav N. Ivanenko, Nicole Bechmann, Yvonne Joseph, Teofil Jesionowski

**Affiliations:** 1 Institute of Experimental Physics, TU Bergakademie Freiberg, Freiberg, Germany; 2 Natural Products Unit, King Fahd Medical Research Center, King Abdulaziz University, Jeddah, Saudi Arabia; 3 Suez Canal University Hospital, Suez Canal University, Ismailia, Egypt; 4 Department of Pharmacognosy, Faculty of Pharmacy, Suez Canal University, Ismailia, Egypt; 5 Department of Natural Products, Faculty of Pharmacy, King Abdulaziz University, Jeddah, Saudi Arabia; 6 Institute of Chemical Technology and Engineering, Faculty of Chemical Technology Poznan University of Technology, Poznan, Poland; 7 Leibniz Institute of Polymer Research Dresden, Dresden, Germany; 8 Clinical Sensoring and Monitoring, Department of Anesthesiology and Intensive Care Medicine, Faculty of Medicine Carl Gustav Carus, Technische Universität Dresden, Dresden, Germany; 9 Department of Prosthetic Dentistry, Faculty of Medicine and University Hospital Carl Gustav Carus of Technische Universität Dresden, Dresden, Germany; 10 P.P. Shirshov Institute of Oceanology of Academy of Sciences of Russia, Moscow, Russia; 11 Department of Invertebrate Zoology, Biological Faculty, Lomonosov Moscow State University, Moscow, Russia; 12 Institute of Clinical Chemistry and Laboratory Medicine, University Hospital Carl Gustav Carus, Faculty of Medicine Carl Gustav Carus, Technische Universität Dresden, Dresden, Germany; 13 Institute of Electronics and Sensor Materials, TU Bergakademie Freiberg, Freiberg, Germany; Tierarztliche Hochschule Hannover, GERMANY

## Abstract

Marine demosponges (Porifera: Demospongiae) are recognized as first metazoans which have developed over millions of years of evolution effective survival strategies based on unique metabolic pathways to produce both biologically active secondary metabolites and biopolymer-based stiff skeletons with 3D architecture. Up to date, among marine demosponges, only representatives of the Verongiida order have been known to synthetize biologically active substances as well as skeletons made of structural polysaccharide chitin. This work, to our knowledge, demonstrates for the first time that chitin is an important structural component within skeletons of non-verongiid demosponges *Acarnus wolffgangi and Echinoclathria gibbosa* collected in the Red Sea. Calcofluor white staining, FTIR and Raman analysis, ESI-MS, SEM, and fluorescence microscopy as well as a chitinase digestion assay were applied in order to confirm, with strong evidence, the finding of α-chitin in the skeleton of both species. We suggest that, the finding of chitin within these representatives of Poecilosclerida order is a promising step in the evaluation of these sponges as novel renewable sources for both biologically active metabolites and chitin, which are of prospective application for pharmacology and biomedicine.

## Introduction

**Fig 1:**
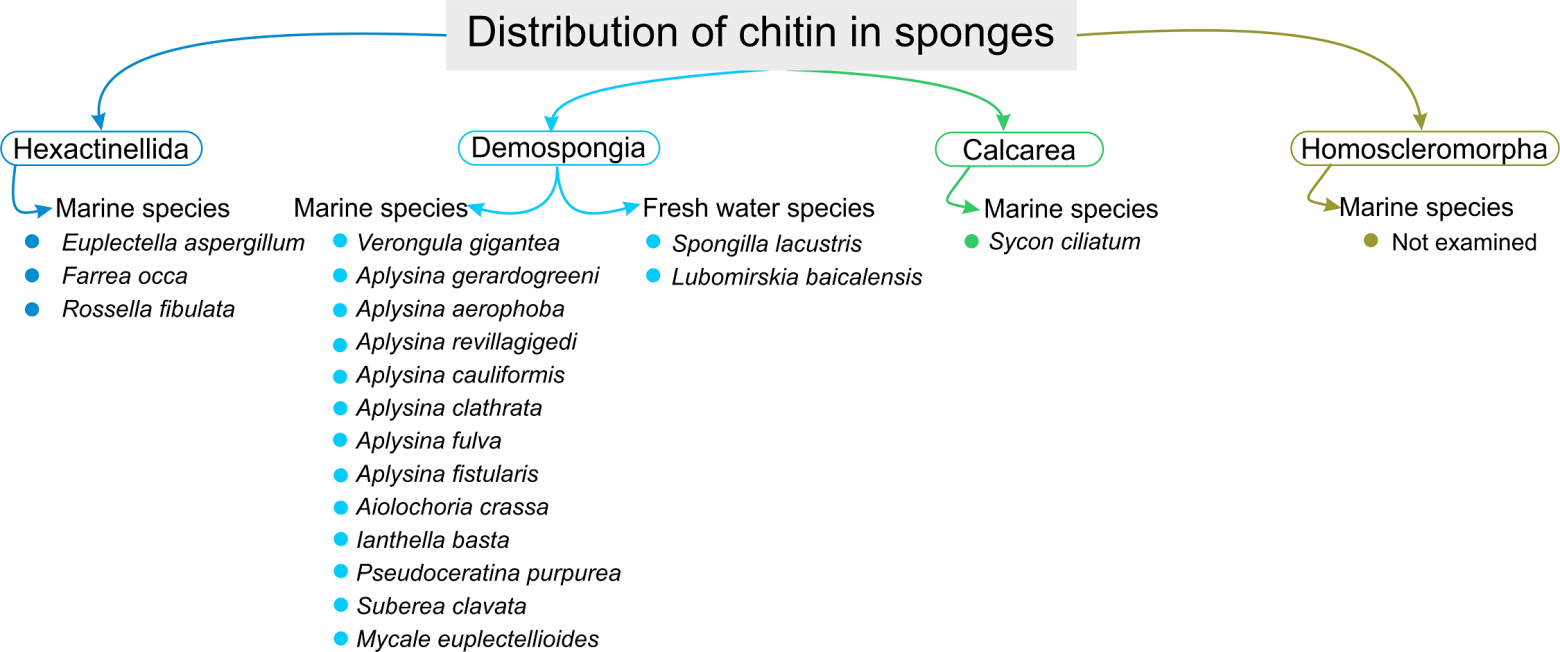
Current state of the art concerning distribution of chitin in the phylum porifera.

Structural aminopolysaccharide chitin is recognized to occur as the basic component in both non-mineralized and mineralized skeletal formations of the cell walls of diverse fungi [1–3], diatoms [4], sponges [5–9], corals [10], annelids [11], molluscs [12,13], and arthropods (see for review [14]). This ancient biopolymer is typically cross-linked due to the complex linkage with pigments, lipids, other polysaccharides, peptides and proteins. As universal template in biomineralization, chitin plays a significant role in formation of calcium- (in molluscs) and silica-based (in diatoms and glass sponges) biominerals [15]. Interaction between diverse organic and inorganic molecules listed above and chitin is often the key way to rigidification of broad variety of skeletal constructs in invertebrates. Mechanical stiffness of skeletons remains to be crucial for surviving of sponges as sessile and filtering organisms. Chitin plays important role in rigidification in some sponges in both non-mineralized and mineralized states have been recently reported in representatives of marine (see for review [16–19]) and fresh water [20,21] sponges. In some demosponges, chitin has been confirmed as template for formation of biomineralized structures in the form of aragonite-silica-chitin composites [22]. Silica-chitin-based skeletal structures have been identified in glass sponges [5,23]. Intriguingly, isolation of chitin-based structures have been never reported in calcarean sponges (class Calcarea), although chitin synthase genes have been already detected in two species [24] (Fig 1). Furthermore, there is no reports in the literature about the existence of chitin in skeletons of the sponges belonging to the class Homoscleromorpha.

Pure chitin, which was traditionally extracted at large scale from fungi and crustaceans, received special attention in the modern industry [25]. The potential of chitin application as adsorbent [26] and biomaterial for biomedical purposes [27] is well known. Recent novel information concerning applied potential of chitin can be found in numerous reviews including few reports [28–30]. It is to note here that industrially produced chitin can be mostly isolated in the form of powders and flakes. Interestingly, sponges originally produce 3D chitinous scaffolds, which are fibrous and macroporous due to their functional role in the skeletons of these filter-feeding invertebrates. This feature has been observed also in the Cambrian fossil demosponges including *Vauxia gracilenta* [31]. Moreover, chitinous skeleton of the demosponges origin resembles the style and form of the source sponges [7]. The nanofibrillar organization together with unique mechanical and thermal properties of chitinous skeletal scaffolds is the key to their successful applications in tissue engineering [9,32,33] and modern biomimetics [34–39]. However, up-to-date this progress has been based exclusively on chitin isolated from diverse representatives of only one order of marine demosponges, the Verongiida order. The idea to propose the use of this feature for systematics of all sponges related to Verongiida order logically appeared. However, our recent findings of chitin in fresh water demosponges [20,21] of non-verongiid origin stimulated the monitoring of chitin also in other demosponges genera, especially in marine species which arose prior to fresh water sponges.

Consequently, two years ago we started intensive monitoring of diverse non-verongiid marine demosponges with the aim to find, purify and characterize and identify chitin from different orders order of marine Demospongiae. Especially, we have taken advantage of the worldwide distribution of the sponges of the order Poecilosclerida which includes four suborders and 25 families. This order is recognized as the largest and most diverse among Demospongiae orders [40] with species occurring in all oceans from shallow water habitats to deep seas.

**Fig 2:**
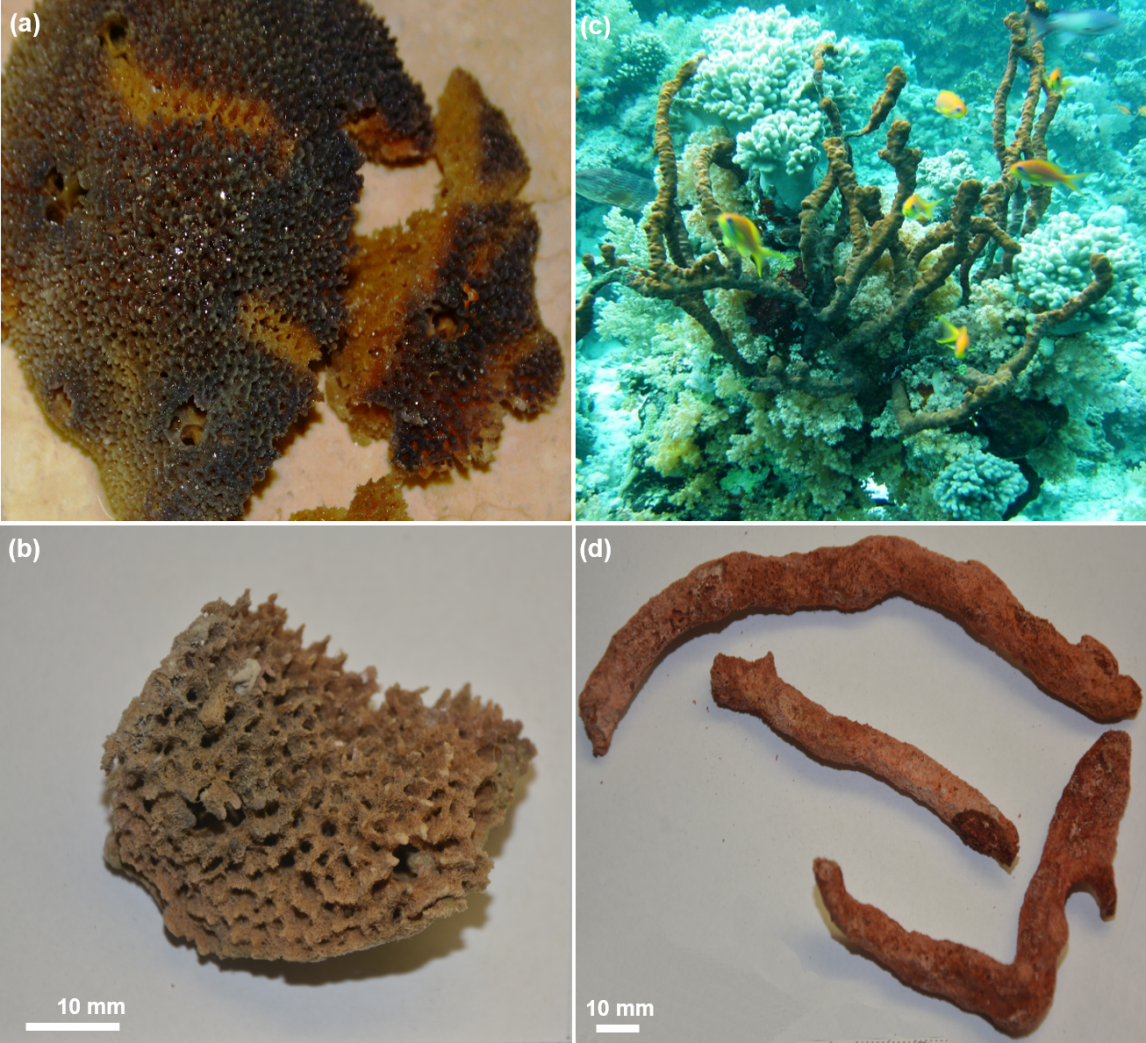
Specimens of *A*. *wolffgangi* (a) and *E*. *gibbosa* (c) in their natural environments. Washed with deionized water of the freeze-dried skeletons of *A*. *wolffgangi* (b) and *E*. *gibbosa* (d) which have been used for chitin isolation in this study.

Preliminary investigations with respect to chitin identification in 60 diverse demosponges recently collected in the Red Sea showed that such representatives of Poecilosclerida as *Acarnus woffgangi* and *Echinoclathria gibbosa* (Fig 2) should contain chitin within their skeletons due to their characteristic insolubility in 2.5 M NaOH solution.

Members of the genus *Acarnus* (Gray, 1867) (Porifera: Poecilosclerida) belong to the family Acarnidae (Dandy, 1922) with 26 representative species [41–45]. *Acarnus wolfgangii* was described for the first time by Conrad Keller in 1889 [46] as a sponge having rigid fiber-based skeleton which show network-like architecture and rich on spongin. The diameter of skeletal fibers was measured as about 0.05 mm and the air dried sponge was stone hard [46].

*Echinoclathria* (Carter, 1885) is a genus of demosponges belonging to the family Microcionidae (Carter, 1885). This family includes two subfamilies, Clathriinae and Ophlitaspongiinae, with nine valid genera and 524 valid species living worldwide in shallow waters with a few records from deeper seas [47]. Unfortunately, with exception of one report on the identification of secondary metabolites from *E*. *gibbosa* [48] no additional reports are available on this species.

**Fig 3:**
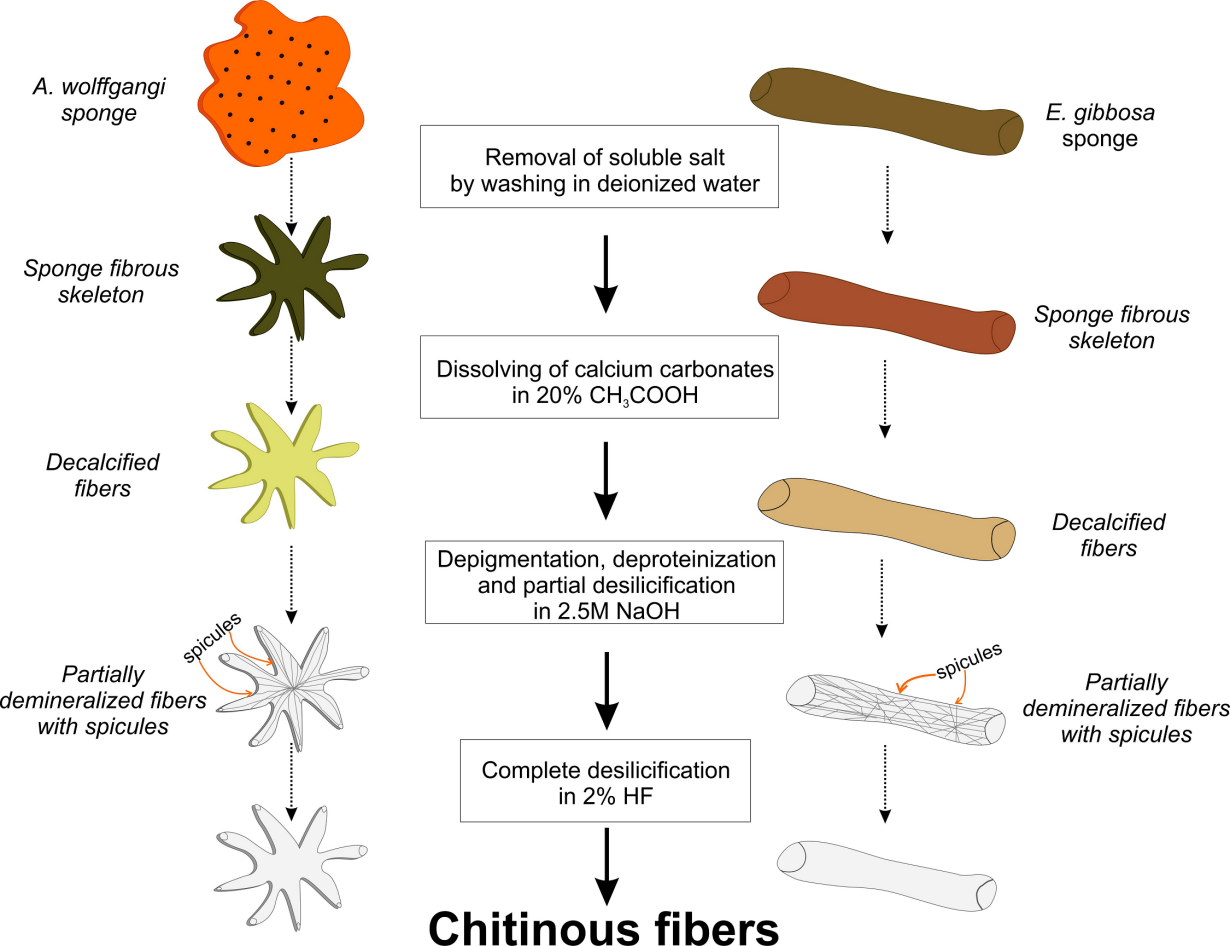
Step-by-step scheme showing procedure for isolation of chitinous fibers from the skeletons of *A*. *wolffgangi* (left line) and *E*. *gibbosa* (right line).

Here, we represent the first study on isolation of chitin from the skeleton of *A*. *wolffgangi* and *E*. *gibbosa* demosponges according to the step-by-step approach (Fig 3) and identification of this structural aminopolysaccharide using corresponding bioanalytical methods in comparative modus.

## Materials and methods

### Biological materials, sample collection and preparation

*Acarnus wolffgangi* Keller (Demospongiae, Poecilosclerida, Acarnidae). The sponges was collected by SCUBA diving in July 2017 from the eastern side of the Small Giftun Island (N 27°11′12.9′′ E 33°59′03.1′′) in the Egyptian Red Sea at a depth of 28 m. The sponge is yellowish in color and forms a massive crust with clathrate surface. The skeleton is formed by a reticulation of skeletal fibers, cored by thick smooth styles and echinated by smooth cladotylotes. The fiber diameter measuring between 50 and 100 μm. At the surface, there are tangentially scattered tylotes. The spicules include: ectosomal tylotes with microspined tyles measuring 215–255 x 2–3 μm, smooth and curved choanosomal styles measuring 300–330 x 15–20 μm and the cladotylotes existing in two distinct shapes and sizes, both with smooth rounded tyles at one end, the larger with three strong hooks at the opposite ends with overall dimensions of 220–290 x 10 μm, and the smaller with four hooks, smooth or occasionally with spined shaft with overall dimensions 90–125 x 3–6 μm. The toxas exist in three distinct categories, including toxas I with oxhorn shape measuring 90–115 x 3–5 μm, toxas II, which is thin with shallow curve measuring 55–65 x 1 μm, and oxea-like toxas III which is barely curved measuring 500–620 x 3 μm; palmate isochelae, 15–20 μm. We were able to study the type material of *Acarnus wolffgangi*, kept in the collections of the Museum für Naturkunde Berlin, ZMB 1498 and 2922. The Red Sea specimen conforms closely in shape, skeleton and spicules, with this type. The Red Sea voucher was kept in the Naturalis sponge collection under registration number ZMA Por. 16636 measuring 10 x 5 by 1 cm in size. Another voucher was kept at the Red Sea Invertebrates Collection at the Department of Pharmacognosy of Suez Canal University under the code number RS-23.

*Echinoclathria gibbosa* (Keller, 1889). The sponge was collected in July 2017 from Hurghada (N 27°17′0.53′′ E 46°22′0.8′′) in the Egyptian Red Sea at a depth of 30 m. The live sponge is blood-red in colour and forms a mass of long branches, which anastomose infrequently. The length of the branches in the voucher sample measuring up to 20 cm with varied thickness due to the irregular outline of the branches from 1 to 2 cm. The surface is pitted and clathrate. The skeleton displayed square- to round-meshed reticulation of skeletal fibers, cored by 1–5 spicules in cross section. The meshes measure 150–300 mm, while connecting fibers measure 10–25 mm in diameter, respectively. The surface skeleton showed a tangential arrangement of loose styles. The spicules are of choanosomal styles and ectosomal subtylostyles, existing in two or three diffeent sizes, but these are not clearly differentiated and measuring about 125–360 x 1–4 mm. The microseleres are very thin, shallow-curved toxas and measuring up to 20 mm in length. The specimen was compared with a slide of the Berlin Museum type and found to conform closely to it. The voucher is registered in the collections of the Netherlands Centre of Biodiversity Naturalis under number ZMA Por. 19793. Another voucher was kept at the Red Sea Invertebrates Collection at the Department of Pharmacognosy of Suez Canal University under the code no. RS-46.

### Isolation of chitin skeleton from *A*. *wolffgangi* and *E*. *gibbosa*

The isolation of chitin-based skeletons from *A*. *wolffgangi* and *E*. *gibbosa* were carried out as previously reported [17–20,49]. The protocol consists of four steps (Fig 3): firstly, the sponge skeletons were placed, separately, in deionized water at room temperature for 1 h in order to remove possible water-soluble sediment particles and salts. Then, the samples were treated with 20% acetic acid at room temperature for 12 h in order to remove residual carbonate-based debris (microfragments of mollusc shells and crustacean carapaces) from the skeleton of *A*. *wolffgangi* and *E*. *gibbosa*. Afterwards, the samples were washed several times with deionized water until achieving a pH of 6.5 followed by treatment with 2.5 M NaOH at 37°C for 72 h to remove pigments and proteins. The siliceous spicules were observed in the samples after 72 h of alkali treatment, thus thorough desilicification was needed. Consequently, after alkali treatment, samples were accurately rinsed with deionized water and placed in a plastic vessel containing appropriate amount of 2% hydrofluoric acid (HF) solution. The vessel was covered in order to prevent the evaporation of HF. Desilicification was conducted at room temperature for 12 h. The effect of alkaline and strong acidic treatments on the structure of skeletons of both demosponges was investigated using optical and fluorescence microscopy (Keyence BZ-8000). Finally, the isolated materials were washed several times with deionized water up to pH of 6.5. The fibrous scaffolds were put into the 250 mL large GLS 80 Duran glass bottles containing deionized water and stored at 4°C for further analyses.

### Light and fluorescent microscopy imaging

Collected sponge samples and isolated chitinous scaffolds from *A*. *wolffgangi* and *E*. *gibbosa* have been observed using BZ-9000 microscope (Keyence) in white light as well as in fluorescence modes.

### Calcofluor White (CFW) test

In order to evaluate the localization of chitin in the demineralized skeleton of *A*. *wolffgangi* and *E*. *gibbosa*, Calcofluor White (Fluorescent Brightener M2R, Sigma) was used as a fluorescent dye for staining of β-(1→3) and β-(1→4) linked polysaccharides [31,49–52]. After binding to polysaccharides containing β-glycosidic bond, such as chitin, this flourochrome emits a bright blue light under UV excitation even using very short light exposure time (up to 1/1000 s) Selected fragments of demineralized skeletons of *A*. *wolffgangi and E*. *gibbosa* were placed in 0.1 M KOH-glycerine-water solution and few drops of 0.1% solutions of CFW were added and the mixture was placed in darkness for 60 min. Afterwards, the stained skeletons were rinsed 5 times with deionized water and dried at room temperature followed by investigation of the scaffolds under fluorescence microscopy.

### Scanning electron microscopy

The surface morphology and microstructure of isolated chitinous scaffolds as well as untreated samples of both sponges were investigated on the basis of SEM images using ESEM XL 30 Philips scanning electron microscope. Before analysis, samples were covered with a carbon layer for 1 min using Edwards S150B sputter coater.

### Raman spectroscopy

Raman spectroscopy was performed using a Raman spectrometer (RamanRxn1™, Kaiser Optical Systems Inc., Ann Arbor, USA) coupled to a light microscope (DM2500 P, Leica Microsystems GmbH, Wetzlar, Germany). For more details, see [17].

The samples displayed intense fluorescence, which made the acquisition of a high quality Raman spectrum impossible. Therefore, the samples were bleached in 10% solution of hydrogen peroxide for 3 h. After three washing steps in distilled water, the samples were dried at room temperature. The Raman spectra were then acquired using an accumulation time of 3 s and summing up 50 accumulations. A baseline correction was finally applied in Matlab to remove the residual fluorescence signal from the spectra and display the Raman scattering.

### Fourier-transformation infrared spectroscopy

FTIR spectroscopy is a powerful tool for the structural analysis of polysaccharides. This method is sensitive to the position and anomeric configuration of glycosidic linkages in glucans. It is worth to note that chitin, depending on its origin and function of tissue, occurs mostly in three main isoforms as α-chitin (fungi, sponges, arthropods), β-chitin (diatoms, molluscs) and rarely as γ-chitin (cocoons of some insects) [14,16]. This vibrational spectroscopy is also sensitive to the geometry of molecules, system of intramolecular and intermolecular interactions. Transmission spectra of chitinous scaffolds were made using a Nicolet 210c FTIR Spectrometer using ATR accessory. The investigation was performed over a wavenumber range of 4000–400 cm^-1^ (at a resolution of 0.5 cm^-1^). The standard α-chitin was purchased from INTIB GmbH, Freiberg, Germany.

### Chitinase digestion test

In order to carry out chitinase digestion test, the Yatalase^®^ enzyme from culture supernatants of *Corynebacterium* sp. OZ-21 (Cosmo Bio, Japan) was used. One unit of this enzyme released 1 μmol of *N*-acetyl-D-glucosamine from 0.5% chitin solution and 1 μmol of p-nitrophenol from p-nitrophenyl-*N*-acetyl-β-D-glucosaminide solution in 1 min at 37°C and pH 6.0. The completely demineralized fibers of *A*. *wolffgangi* and *E*. *gibbosa* were incubated in enzyme solution containing 10 mg Yatalase dissolved in 1 mL of citrate phosphate buffer at pH 5.0 for 2 h. The effectiveness of enzymatic digestion was monitored using optical microscopy (Keyence).

### Estimation of *N*-acetyl-D-glucosamine (NAG) contents (Electrospray ionization mass spectrometry ESI-MS)

The Morgan–Elson assay was used in order to estimate the *N*-acetyl-D-glucosamine content released after chitinase treatment, as previously reported [17,20].

Preparation of the samples for ESI-MS: the demineralized organic scaffolds of *A*. *wolffgangi* and *E*. *gibbosa* were hydrolysed in 6 M HCl for 24 h at 50 ^o^C. After hydrolysis samples were filtrated with 0.4 μm filter and freeze-dried to remove the excess of HCl. The dried samples were dissolved in deionized water for analysis. All ESI-MS measurements were performed on Waters TQ Detector ACQUITYuplc mass spectrometer (Waters, USA) equipped with ACQUITYuplc pump (Waters, USA) and BEHC18 1.7 μm, 2.1 × 50 mm UPLC column. Nitrogen was used as nebulizing and desolvation gas. Graphs were generated using Origin 8.5 for PC.

## Results

**Fig 4:**
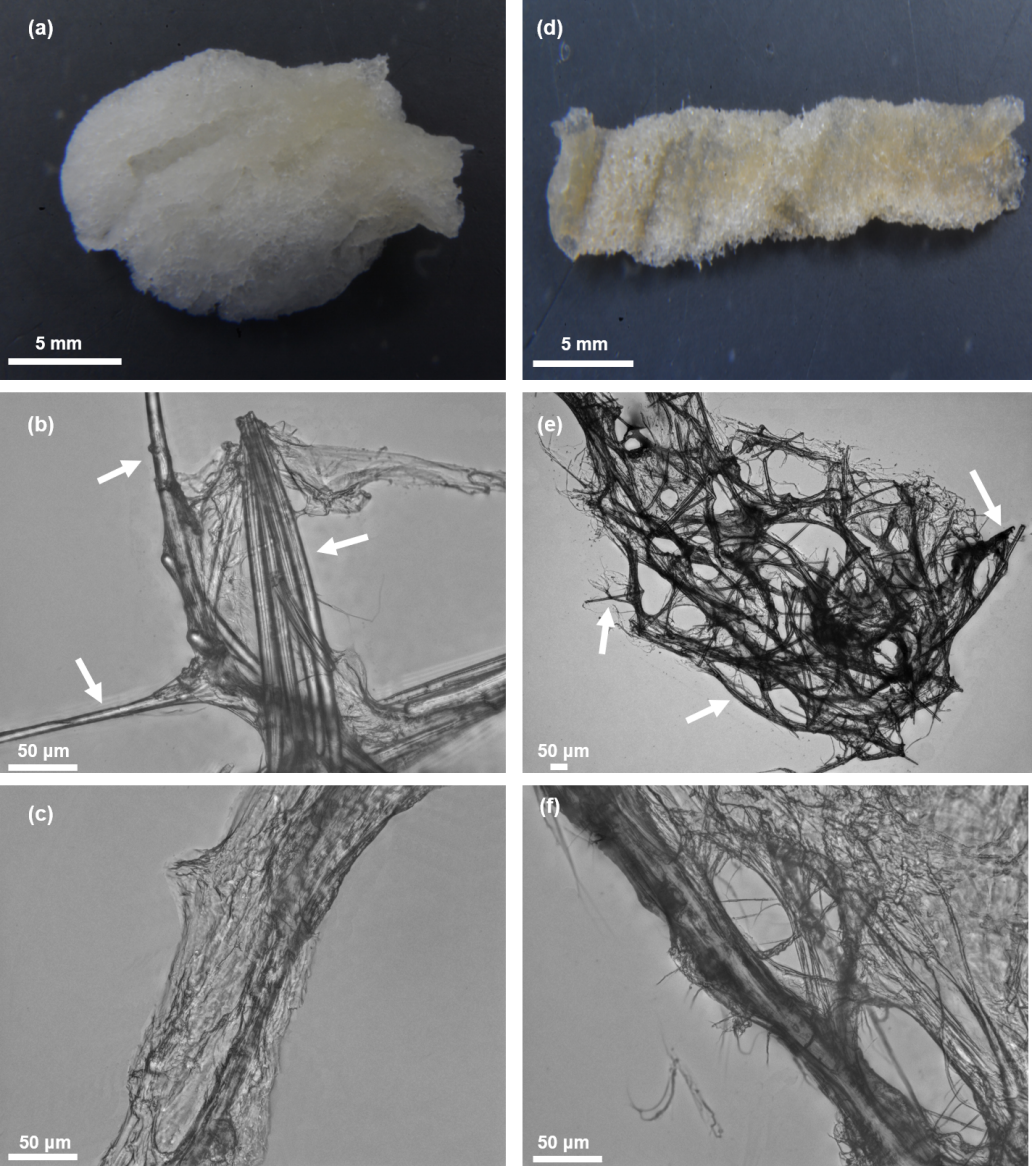
Spicule-free, colorless 3D scaffold obtained from *A*. *wolffgangii* (a) and *E*. *gibbosa* (d) according to the isolation procedure represented in Fig 3. Microstructural features of selected skeletal fibers of *A*. *wolffgangii* (marked with arrows) (b) and *E*. *gibbosa* (e) prior and after HF-treatment (c and f, respectively) are well visible on the corresponding light microscopy images.

Fig 4 clearly indicates that the applied chemical treatment procedures (detailed presented in Fig 3) lead to purification of the fibrous scaffolds with well-organized anastomosing morphology from the skeletons of *A*. *wolffgangi* and *E*. *gibbosa*, respectively. The images presented in Fig 3A and 3D show that the overall shape and morphology of the extracted 3D scaffolds closely resemble the styles and forms of the investigated sponges (Fig 2B and 2D). This means that, the isolation procedure does not lead to a breakdown of the–sometimes very fragile–demosponge structures, even after HF-based removal of the skeleton supporting spicules.

**Fig 5:**
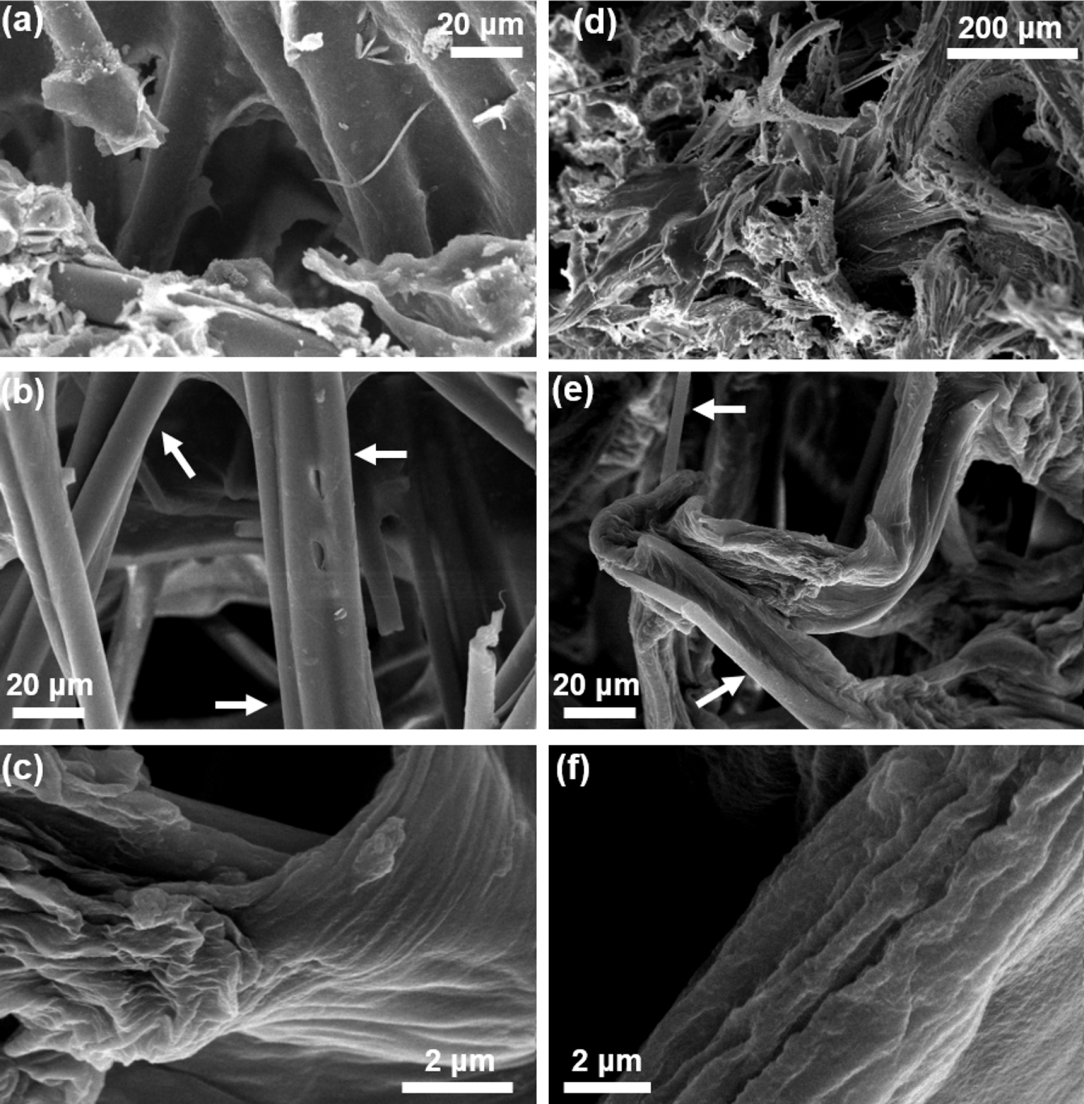
SEM imagery of the purified *A*. *wolffgani* (a) and *E*. *gibbosa* (d) skeleton’s fragments prior (b and e, respectively) and after demineralization procedure (c and f, respectively). Well visible spicules are marked with arrows.

SEM microphotographs of the skeletal fibers of *A*. *wolffgangi* and *E*. *gibbosa* prior to any treatment confirmed the complex character of their skeletons where, various forms of inorganic (spicules) as well as organic (fibres) structures are well visible. Fig 5B and 5E) show that glassy spicules were still present within skeletal scaffolds isolated from both sponges after NaOH and acetic acid treatment. Only HF-based treatment leads to dissolution of the spicules and purification of silica-free microfibers (Fig 5C and 5F).

**Fig 6:**
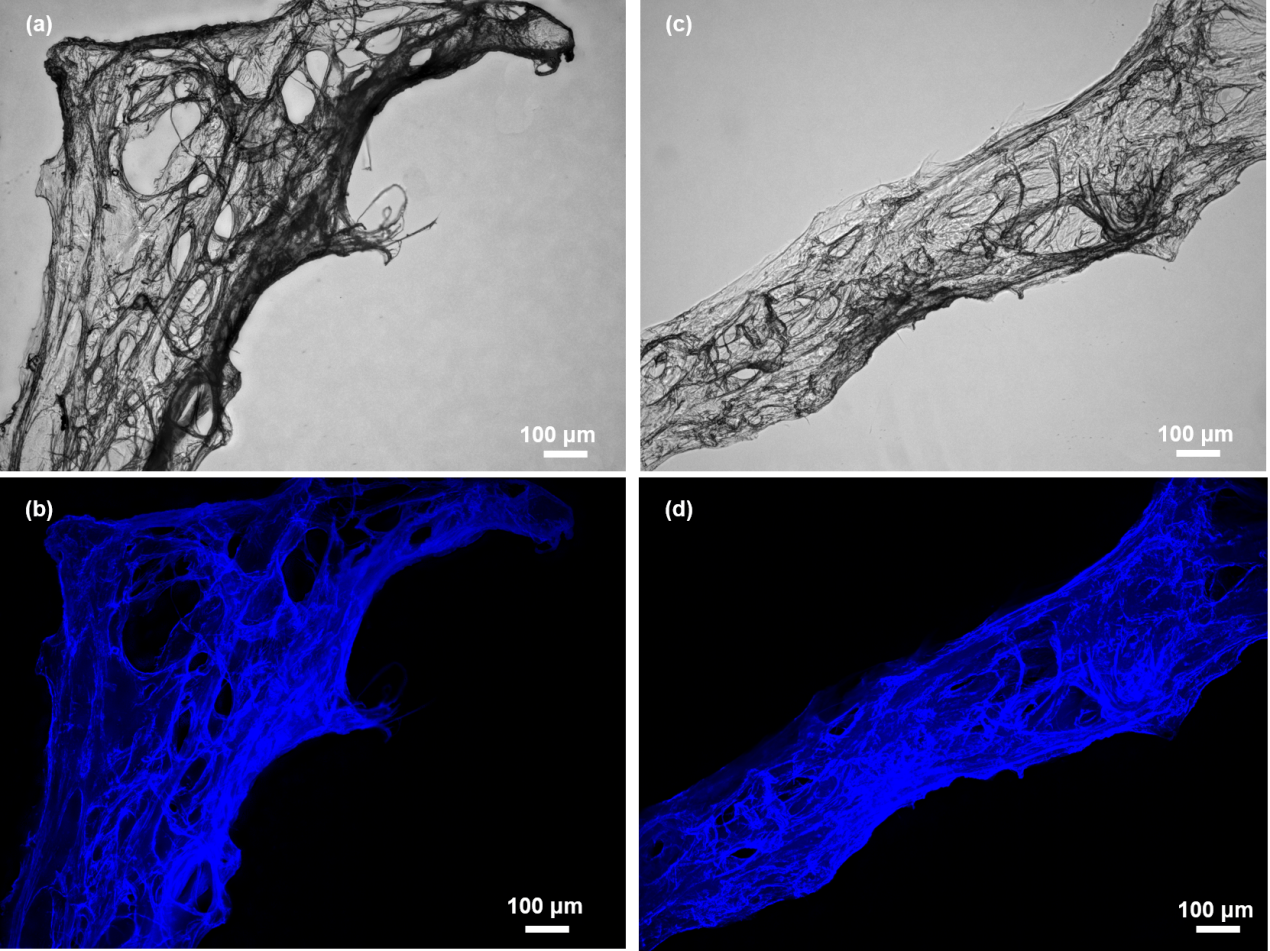
Purified skeletal fibers of *A*. *wolffgangi* (a) and *E*. *gibbiosa* (c) after CFW staining observed in light microscopy (a, c) and fluorescence microscopy (b and d) modus, respectively.

The Calcofluor white staining (CFW) [50] was the first step for the preliminary identification of chitin within isolated and demineralized skeletal samples. Fluorescence microscopy analysis of the scaffolds isolated from *A*. *wolffgangi* and *E*. *gibbosa* after CFW staining displayed very strong fluorescence even under light exposure time as short as 1/4800 s (Fig 6B and 6D). Corresponding results were previously reported for chitin isolated from marine [7,17,18] and freshwater sponges [20] as well as in chitin-containing fossilized remnants [31,51].

**Fig 7:**
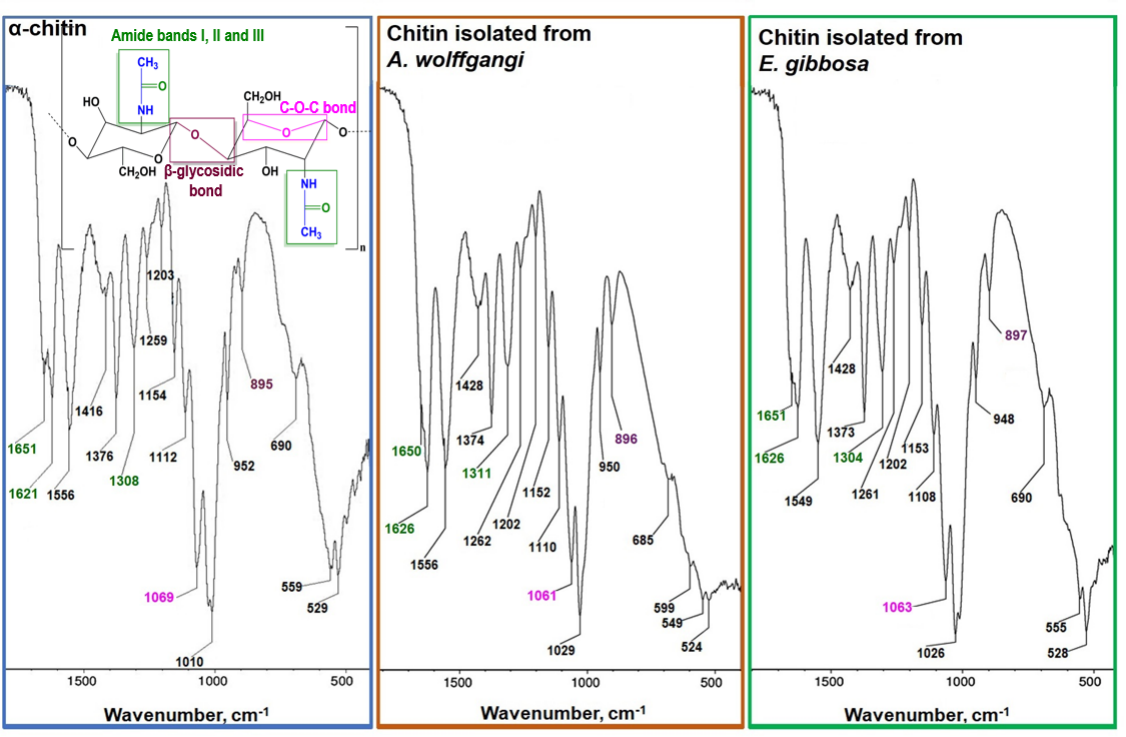
FT-IR spectra of chitin isolated from *A*. *wolffgangi* and *E*. *gibbosa* demosponges in comparison with the of α-chitin standard.

To confirm the presence of chitin in the isolated scaffolds (Fig 4A and 4D), more sensitive analytical techniques were applied. FTIR spectra acquired for the fibrous scaffolds obtained from *A*. *wolffgangi* and *E*. *gibbosa*, as well for α-chitin standard are presented in Fig 7. The region of the amidic moiety, between 1700 and 1500 cm^−1^, yields different signatures for chitin polymorphs. In this region, the spectra of the samples studied by us showed strong adsorption band associated with the stretching vibrations of C = O group characteristic of the amide band I. The amide band I showed twin peak at 1659 cm^-1^ and 1626 cm^-1^ for *A*. *wolffgangi*; 1659 cm^-1^ and 1626 cm^-1^ for *E*. *gibbosa*, as a result of the intermolecular C = O⋯H-N and the intramolecular hydrogen bonds C = O⋯HO-CH_2_ which is characteristic for α-chitin polymorph [53,54]. Additional feature, the characteristic intense band at 950 cm^−1^ assigned to γCHx was observed in α-chitin standard as well as in the purified sponges chitin samples. Moreover, the α-chitin indicative band assigned to a ß-glycosidic bond is observed at a *ν*_*max*_ 897 cm^−1^ in the FTIR spectra of the scaffolds isolated from *A*. *wolffgangii* and *E*. *gibbosa* (Fig 7). Detailed analysis of the bands indicates that acquired spectra of both isolated chitinous scaffolds are very similar to those of the α-chitin standard.

**Fig 8:**
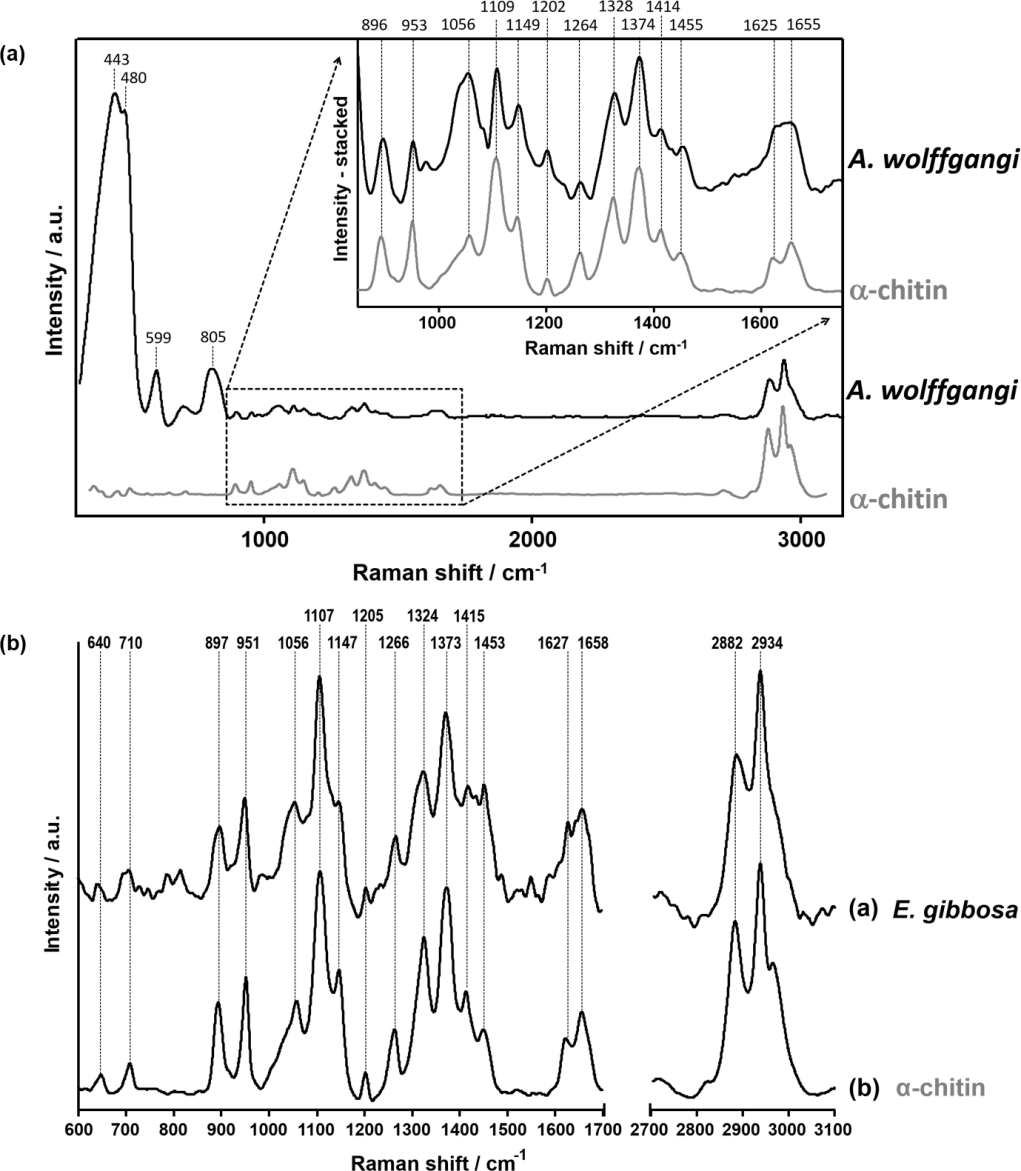
Raman spectroscopy of the chitinous scaffolds isolated from *A*. *wolffgangi* and *E*. *gibbosa* demosponges in comparison with *α*-chitin standard.

The results of Raman spectroscopy examinations showed that spectra of *A*. *wolffgangi* and *E*. *gibbosa* are very comparable with the spectrum obtained for α-chitin reference (see Fig 8). For analytical investigations of the isolated scaffolds, prior and after HF-treatment, Raman spectroscopy was used. Consequently, for example, the Raman spectra of chitinous scaffold isolated from *A*. *wolffgangi and E*. *gibbosa* prior to demineralization display intense bands of biosilica at 443, 480, 599, 640, 805 cm^-1^. The bands of the organic matrices are visible in the ranges ≈ 900–1800 cm^-1^ and 2700–3000 cm^-1^. These bands are comparable with those reported for α-chitin standard. Similar observations have been reported for chitin of demosponge origin previously [5–9,18,49].

**Fig 9:**
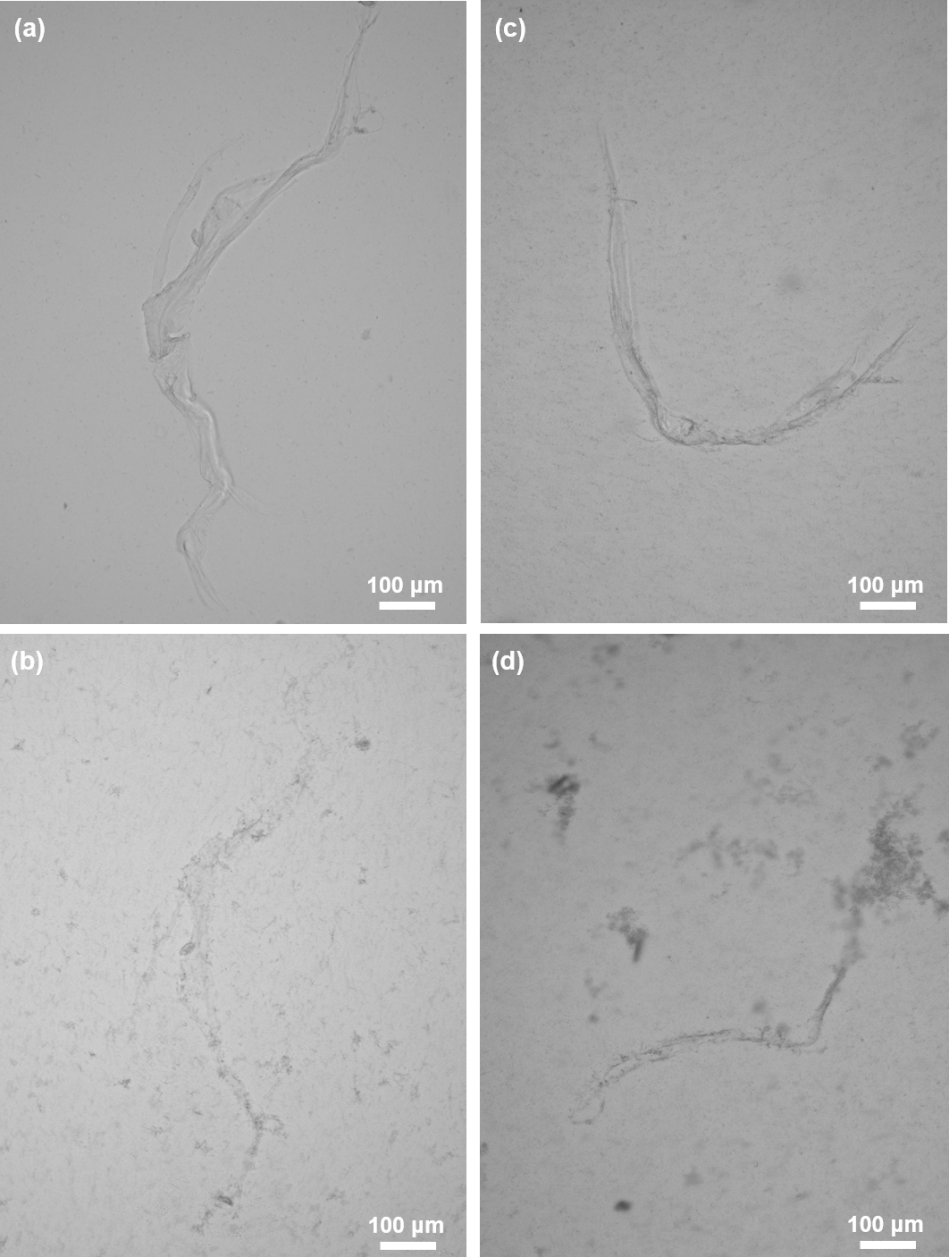
Visualization of the chitinase digestion test using white light microscopy. Chitinase digestion of purified and completely demineralized selected skeletal fiber isolated from *A*. *wolffgangi* and *E*. *gibbosa* prior (a and c, respectively) and after 3 h of chitinase treatment (b and d, respectively).

Chitinases possess the ability to degrade chitin directly to low molecular weight chitin oligomers including *N*-acetylglucosamine (GlcNAc). Consequently, such enzymatic treatment resulted in the loss of chitin integrity and in release of residual chitin microfibers of steadily decreasing size. The activity of chitinase is clearly visible using an optical microscope (Fig 9). Chitinase digestion test which have been previously utilized in the studies for the chitin detection in other sponges [5–9,17,49,55], definitely confirmed the chitinous nature of demineralized scaffolds isolated from both *A*. *wolffgangi* and *E*. *gibbosa*.

The Morgan–Elson assay has been previously described in details [6,20] and was used as the most accurate methods to estimate the GlcNAc released after chitinase treatment. Determination of GlcNAc in chitin-based scaffolds of *A*. *wolffgangi and E*. *gibbosa* showed, 750 ± 1.5 μg and 730 ± 1.5 μg *N*-acetyl-glucosamine per mg of chitinous scaffolds of these sponges, respectively. These results are similar to those reported for chitin isolated from the demosponge *Spongilla lacustris* [20].

**Fig 10:**
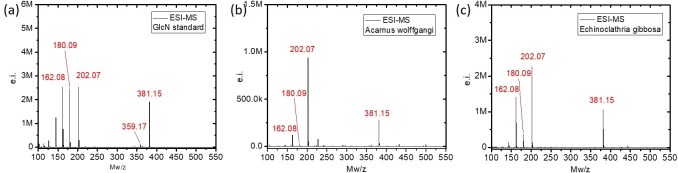
Comparative ESI-MS analyses from the glucosamine standard (a), and of the hydrolysed chitin from the *A*. *wolffgangi* (b) and *E*. *gibbosa* (c).

ESI-MS of D-glucosamine (GlcN) standard showed four main peaks at m/z = 162.08, 180.09, 202.07 and 381.15 (Fig 10). The ion peak with m/z = 180.09 corresponds to the molecular ions [M+H]^+^ of a species with a molecular weight 179.09 corresponding to GlcN (calculated: 179.1). The ion peak at m/z = 162.08 corresponds to a fragment ion [M−H_2_O + H]^+^ after losing one molecule of H_2_O from DGlcN (calculated: 162.1). Finally, the ion peak at m/z = 381.15 corresponds to [2M+Na]^+^ species which is sodium-bound GlcN non covalent dimmer. Similar ion peak for the proton-bound GlcN covalent dimmer was observed at m/z 359.17 [M+H]^+^ in the spectra.

The major peaks in ESI-MS spectra of the hydrolyzed samples of *A*. *wolffgangi* and *E*. *gibbosa* observed at m/z = 162.08, 180.09, 202.07 and 381.15 are comparable with the peaks of GlcN (Fig A in S1 File). The ion peak of the sodium-bound GlcN dominated the spectra of these sponges as expected for marine-derived samples due to strong salt presence [31].

## Discussion

Previously, members of the genus *Acarnus* have been mostly investigated as a source of pharmacologically active compounds. For example, a group of compounds named acarnidines were isolated by extraction of the homogenized tissues of the sponge *A*. *erithacus* with toluene-methanol (1:3) and partitioning with 1 M sodium nitrate solution. They possess unique substituted homospermidine skeleton with diverse fatty acid substituents. The acarnidines showed antibacterial and antifungal properties and displayed significant antiviral activity against Herpes simplex type 1 [56]. Two cyclic peroxide-containing polyketide C22 methyl esters, peroxyacarnoic acid methyl esters A and B, have been isolated from the Red Sea marine sponge *Acarnus cf*. *bergquistae* [57]. The methanolic extract of this sponge exhibited cytotoxicity against P-388, A-549, and HT-29 tumor cells with an IC_50_ of 0.1 μg/ml [58]. Two new cyclic peroxides have been reported in the organic extract of the sponge *A*. *bicladotylota* from India [59].

Furthermore, sponges of the genus *Echinoclathria* are recognized as producers of biologically active compounds. Azaspiracid-2 was isolated from a marine sponge *Echinoclathria* sp. collected off Amami-Oshima area in Japan. It exhibited potent cytotoxicity against P388 cells with an IC_50_ value of 0.72 ng/mL and caused S phase arrest on the cell cycle [60]. The demosponge *E*. *subhispida* gave a new steroid sulfate, echinoclasterol sulfate with experimentally confirmed antifungal activity against *Mortierella ramannianus*, and cytotoxicity against PC-9 human lung cancer cells [61]. Echinoclathrines A-C, a new class of pyridine alkaloids possessing a 4-aryl-2-methylpyridine moiety as a common structural element were isolated from an Okinawan sponge *Echinoclathria* sp [62]. The procedure of the isolation of echinoclathrines have been patented recently [63]. Some of echinoclathrines exhibited weak immunosuppressive activity in a mixed lymphocyte reaction assay. Studies on marine pharmacology potential of *Echinoclathria* demosponges habituated in Red Sea started only recently. Investigation of the Red Sea sponge *E*. *gibbosa* resulted in the isolation of three new compounds including β-sitosterol-3-O-(3*Z*)-pentacosenoate, 5α-pregna-3β-acetoxy-12β,16β-diol-20-one, and echinoclathriamide together thymine and uracil [48]. β-Sitosterol-3-O-(3*Z*)-pentacosenoate showed weak activity against A549 non-small cell lung cancer (NSCLC), U373 glioblastoma (GBM), and PC-3 prostate cancer cell lines [48]. New ceramide (icosanamide) was isolated from the Red Sea sponge *Echinoclathria* sp. [64]. The *in vitro* growth inhibitory activity of this ceramide against different human cancer cell lines was evaluated.

To our best knowledge there are no reports even about attempts to search for chitin in these species of demosponges. Till now, isolation protocols of diverse secondary metabolites from representative members of the genera *Acarnus* and *Echinoclathria* have followed traditional organic solvent-based extraction approaches. There are no data on isolation methods for such metabolites which are based on treatment with alkaline solutions as well as about structural stability of these biomacromolecules at alkaline pH levels. It is well known that, since the experimental work done by von Kölliker in 1864 [65] the main skeletal protein of demosponges-spongin is quickly soluble in alkali solutions [66,67]. This feature is crucial for extraction and isolation of poriferan chitin in purified form due to its exceptional resistance to the treatment with alkali up to concentration of 5% and temperatures not higher than 40°C for example, in the case of NaOH [5,6,8,9,20,21,31,34]. Such treatment showed also no electron microscopically visible changes on the surface of siliceous spicules of the demosponges under investigation (see Fig 5). Moreover, our observations showed with convincing support the localization of spicules within chitinous (Figs 3 and 5) and non-spongin based matrix. Similar results have been reported before in the case of chitinous skeleton of the fresh water demosponge *S*. *lacustris* (order Spongillida) [20]. The possible role of poriferan chitin as structural support for spicule-producing cells as well as in complete process of spiculogenesis in demosponges is still unknown.

**Fig 11:**
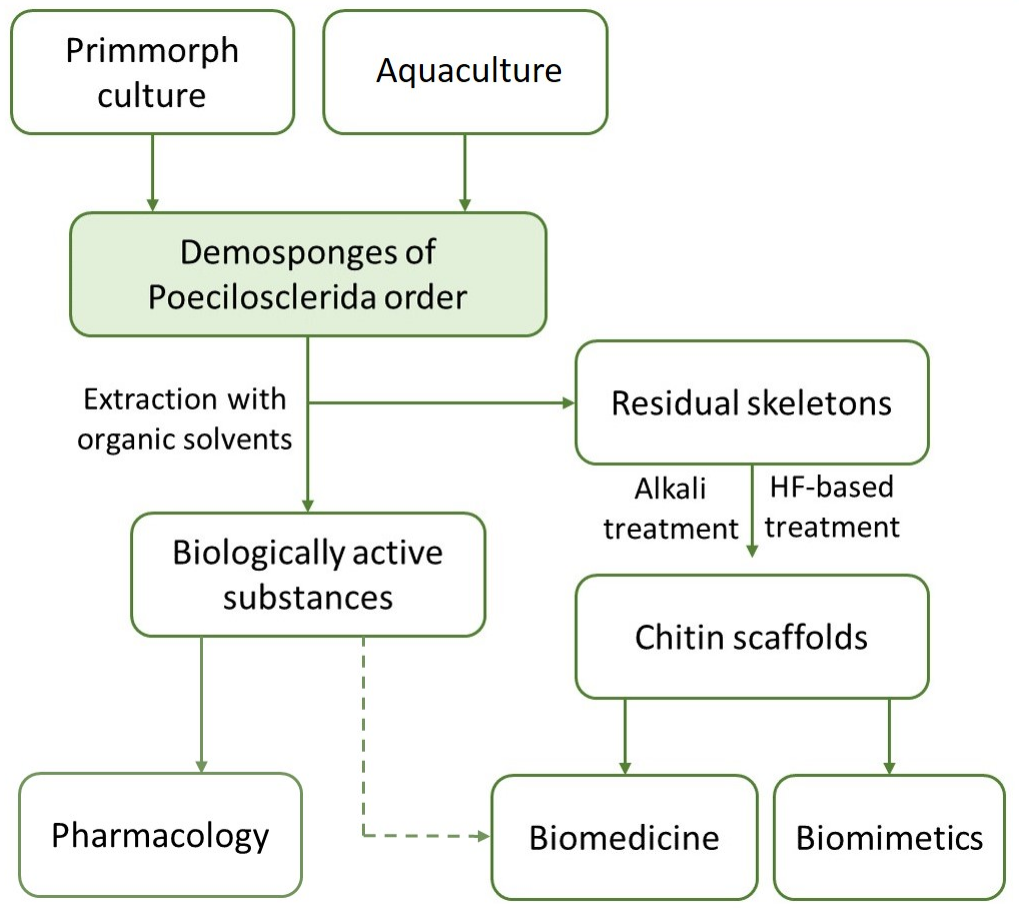
Schematic view of the possible uses of Poecilosclerida sponges including *A*. *wolffgangi* and *E*. *gibbosa* species.

Complete desilicification of the spicules can be achieved using HF-based treatment. This study together with previously reported data [7,18,19,20,21] showed that chitin remains to be well preserved even after such treatment. However, logical question about the possible presence of diverse secondary metabolites with alkaline, or HF-based extracts remain to be open. In contrast, the experiments with bromotyrosine- and chitin-producing demosponges representing the order Verongiida showed that bromotyrosines and chitin-based scaffolds could be isolated from the sponge skeletons using a stepwise extraction procedure mainly based on the use of NaOH [6]. Recently, a patented method for isolation of both bromotyrosines and chitinous skeletal frameworks from selected sponges, without disruption of the skeletons in the mortar has been reported [68]. Here, we propose a schematic view of the principal steps which can be now applied for isolation of secondary metabolites and chitin from the sponges of the order Poecilosclerida (Fig 11).

There are no doubts about the necessity for the development of novel, more effective technologies for extraction of biologically active compounds together with chitinous scaffolds from sponges of the genera *Acarnus* and *Echinoclathria*. Especially those species which could be adapted for cultivation under marine farming conditions will possess high potential in this case.

We suggest that the discovery of chitin within other representatives of Poecilosclerida order would be the next step in the evaluation of the possibility to accept these worldwide distributed demosponges as novel renewable source for both chitin and biologically active metabolites which are perspective for biomedicine and marine pharmacology, respectively.

## Conclusions

Chitin-producing marine demosponges are highly perspective invertebrates due to their ability to synthetize broad variety of secondary metabolites with antiviral, antibiotic, antidiabetic, cytotoxic and antitumor activities as well as chitin. Here, we showed for the first time that chitin is present as a structural component in skeletons of the Red Sea sponges *Accarnus wolffgangi* and *Echinoclathria gibbosa* demosponges. The question of chitin synthesis among representatives of the genera *Acarnus* and *Echinoclathria* should gain importance as a result of our findings. Consequently, the evolution, localization and functions of chitin in these demosponges as well as in other representatives of Poeciloscrerida order should be examined in the future. Additionally, separate studies should be carried out on the identification of chitin synthase genes within genomes of diverse representatives of the genera *Acarnus* and *Echinoclathria* as well. Also, additional investigations are necessary to obtain a better understanding of the nature and origin of spicules-containing skeletons of these demosponges with respect to the spongin-chitin relationship. It is still unclear how much spongin is present in the chitin-based skeletons of the sponges studied. Novel approaches must be proposed which will bring together molecular biology and modern bioanalytical methods for a better understanding of the poriferan chitins synthesis in diverse taxa on molecular level. The best way to solve this challenging task is to bring together coherent and synergetic collaborators and experts in marine biology, marine chemistry, marine pharmacology, marine biotechnology and biomaterials together with spongologists using their multidisciplinary knowledge and experiences to answer raised questions and develop new approaches in this interesting area of research.

## Supporting information

S1 File Shows detailed descriptions of analytical high performance liquid chromatography–mass spectroscopy analysis (LCMS). (DOCX)
