# Supplementary material for: Discovery of chitin in skeletons of non-verongiid Red Sea demosponges
Source: PLoS One. 2018 May 15;13(5):e0195803. doi: 10.1371/journal.pone.0195803 (PMC5953452; doi:10.1371/journal.pone.0195803)
Supplement: S1 File — (DOCX) [file pone.0195803.s001.docx]

**S1 File: Analytical high performance liquid chromatography – mass spectroscopy analysis (LCMS):**

HPLC experiments were performed by analytical HPLC Luna C-18 column (5 μm particle size, 3×250 mm; Waters, Milford, USA). Water containing 0.1 % (v/v) trifluroacetic acid was used as the mobile phase. ESI-MS measurements were performed on Agilent Technologies 6230 TOF LC/MS spectrometer (Applied Biosystems, USA). Nitrogen was used as nebulizing and desolvation gas.


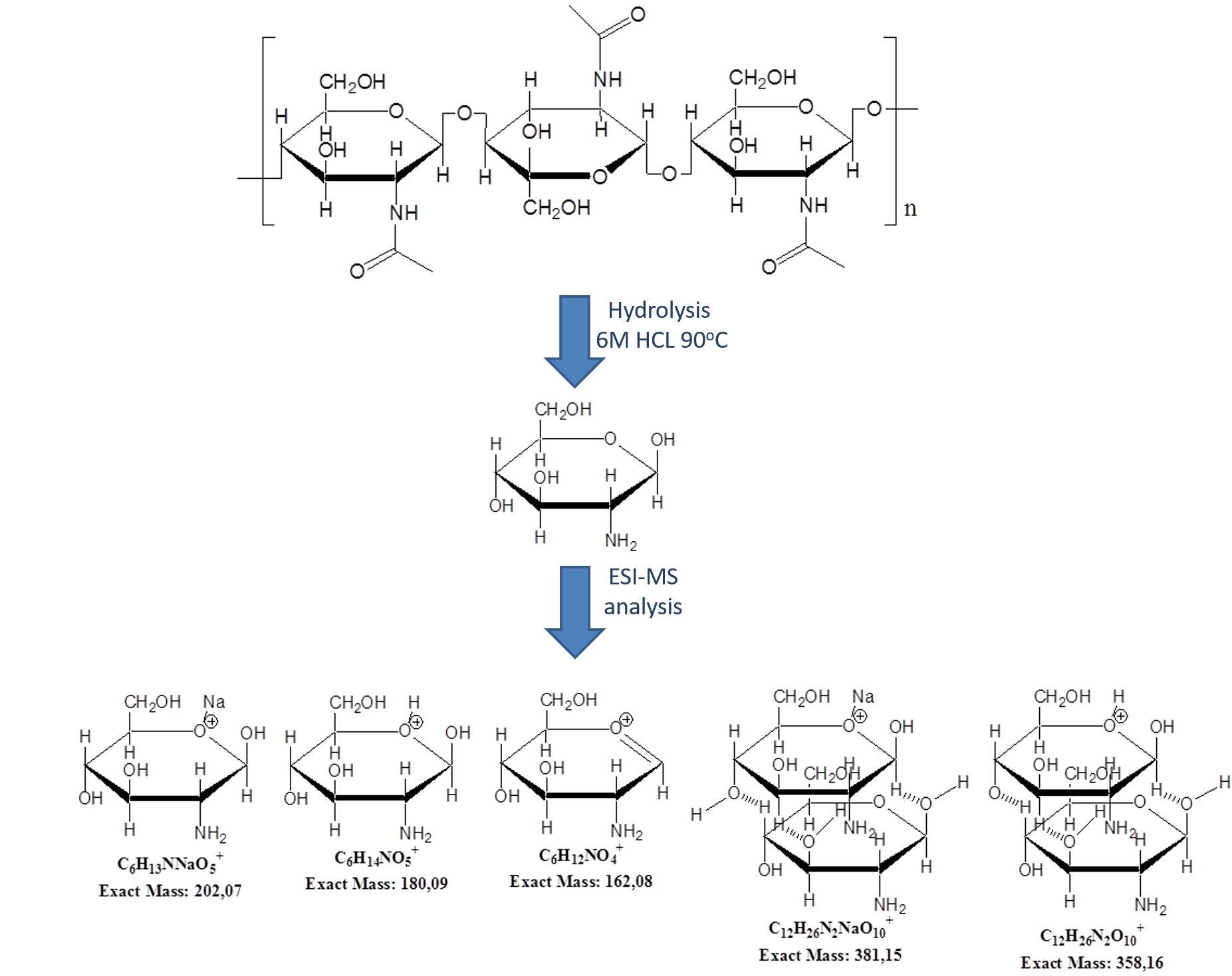


**Figure S1** Schematic view of analysis of chitin hydrolysis by LCMS.
